# Dopamine in Motor Cortex Is Necessary for Skill Learning and Synaptic Plasticity

**DOI:** 10.1371/journal.pone.0007082

**Published:** 2009-09-17

**Authors:** Katiuska Molina-Luna, Ana Pekanovic, Sebastian Röhrich, Benjamin Hertler, Maximilian Schubring-Giese, Mengia-Seraina Rioult-Pedotti, Andreas R. Luft

**Affiliations:** 1 Clinical Neurorehabilitation, Department of Neurology, University of Zurich, Zurich, Switzerland; 2 Department of Neurosciences, Brown University, Providence, Rhode Island, United States of America; 3 Division of Brain Injury Outcomes, Department of Neurology, Johns Hopkins University, Baltimore, Maryland, United States of America; Victoria University of Wellington, New Zealand

## Abstract

Preliminary evidence indicates that dopamine given by mouth facilitates the learning of motor skills and improves the recovery of movement after stroke. The mechanism of these phenomena is unknown. Here, we describe a mechanism by demonstrating in rat that dopaminergic terminals and receptors in primary motor cortex (M1) enable motor skill learning and enhance M1 synaptic plasticity. Elimination of dopaminergic terminals in M1 specifically impaired motor skill acquisition, which was restored upon DA substitution. Execution of a previously acquired skill was unaffected. Reversible blockade of M1 D1 and D2 receptors temporarily impaired skill acquisition but not execution, and reduced long-term potentiation (LTP) within M1, a form of synaptic plasticity critically involved in skill learning. These findings identify a behavioral and functional role of dopaminergic signaling in M1. DA in M1 optimizes the learning of a novel motor skill.

## Introduction

Levodopa, a stable precursor of dopamine (DA), improves the recovery of movement abilities in disabled stroke survivors when given by mouth before daily physiotherapy sessions [1]. Plastic adaptations in the brain are partly responsible for such recovery processes [2], [3] as well as for learning of a motor skill [4]. Levodopa improves such adaptations in stroke [5] and healthy subjects [6], and its metabolite levels correlate with the effectiveness of skill learning [7]. Finally, skill learning is impaired in patients with Parkinson's disease in whom the brain's dopaminergic system degenerates [8]. The mechanisms for all these phenomena are unknown.

Here, we present findings suggesting that these phenomena are explained by dopaminergic transmission in primary motor cortex (M1) that enables M1 synaptic plasticity.

Midbrain dopaminergic neurons project diffusely to many cortical regions, among those to primary motor cortex [9]. Dopaminergic terminals have been demonstrated in superficial and deep layers of the rodent and primate M1 [9], [10]. DA receptors of the D1 and D2 subtype are present in M1 in these species [11], [12], [13] as well as in humans [14]. The functional role of this projection for behavior is unknown.

In prefrontal cortex (PFC) and striatum DA is involved in the expression of synaptic plasticity by modulating the amount of long-term potentiation (LTP) and depression (LTD) of glutamatergic synapses [15], [16], [17], [18], [19]. LTP and LTD are the best characterized candidate mechanisms underlying learning and memory [20], [21]. In M1, neurons are capable of expressing LTP and LTD [22]. Learning a new motor skill exhausts the capacity of M1 neurons to express LTP [23] suggesting that M1-LTP is a cellular mechanism enabling motor skill learning [24], [25], [26].

We therefore hypothesized that dopaminergic neurotransmission in M1 supports motor skill learning by modulating M1 synaptic plasticity. We show that pre- and postsynaptic interference with dopaminergic neurotransmission in M1 impairs motor skill learning and M1 synaptic plasticity.

## Results

### Dopamine terminals in M1 and motor skill learning

**Figure 1:**
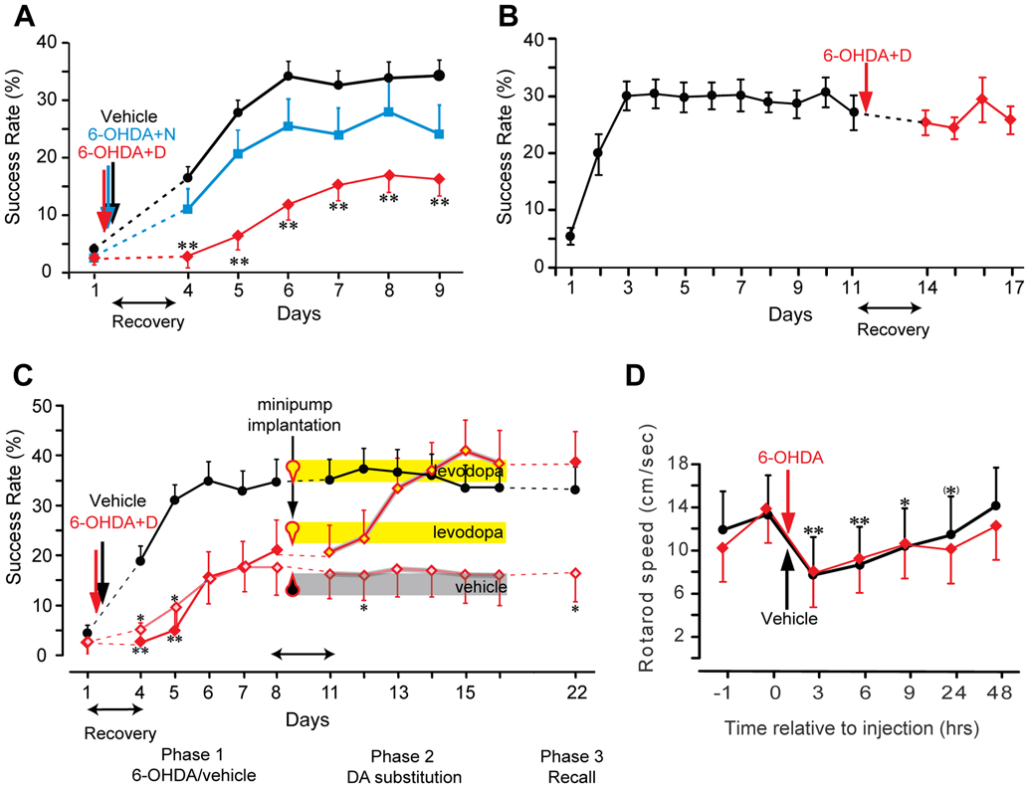
Dopamine (DA) release in M1 is necessary for optimal motor skill acquisition but not for movement execution. (a) Learning curves for sham-lesioned rats (black, vehicle), rats with dopaminergic terminals destroyed (red, 6-OHDA+D), and rats with noradrenergic terminals destroyed (blue, 6-OHDA+N). Cortical injections (vertical arrows) were performed following an initial training session to determine paw preference. After 3 days of recovery from surgery (horizontal arrow, necessary interval determined in d) rats were trained for 6 successive days. The success rate of skill acquisition was significantly impaired in animals with without dopaminergic terminals but not in animals without noradrenergic terminals (** p<0.05). (b) DA is not required for task performance because elimination of dopaminergic terminals in M1 (red, vertical arrow) in rats that already acquired the reaching skill (black) did not affect reaching performance. (c) Learning impairment is restored with DA substitution (administration of its precursor levodopa). Rats received cortical injections of 6-OHDA+D and were trained comparable to a): As compared with sham-lesioned animals (vehicle-injected, black), the two groups without dopaminergic terminals in M1 (6-OHDA+D-injected, red) demonstrated a learning impairment – *phase 1*. Rats were then implanted with minipumps (drops): 50% of DA terminal deficient rats received vehicle (grey) and 50% received levodopa (yellow) during the entire second training period, sham-lesioned rats received levodopa – *phase 2*. Learning was restored in DA-substituted rats underlining the importance of DA for skill acquisition. Minipumps were then removed and all rats were examined for task recall after 6 days of rest. DA is not required to recall an already learned skill as indicated by unchanged performance levels in all groups. (d) Cortical injections independent of whether 6-OHDA or vehicle was used transiently impair locomotor function. Rotarod tests were performed in vehicle- (black) and 6-OHDA (red) injected rats. Parallel deficits indicate that reduced rotarod speed results from injection or surgery and not from the drug itself. Results were used to determine the recovery period following surgery (horizontal arrows in a-c).

Motor skill learning was impaired in rats with destroyed M1 dopaminergic terminals in the forelimb area of the primary motor cortex (M1). Dopaminergic terminals were selectively eliminated by intracortical injection of 6-hydroxydopamine (6-OHDA) into the M1 forelimb area of the hemisphere contralateral to the preferred forelimb in conjunction with desipramine (i.p.) to protect noradrenergic terminals (6-OHDA+D, n = 6). The location of the forelimb area was determined by stimulation mapping. Learning curves were compared to sham-lesioned rats (vehicle, n = 11) and to rats with selectively destroyed M1 noradrenergic terminals by intracortical injection of 6-OHDA into the M1 forelimb area in conjunction with nomifensine (i.p.) to protect dopaminergic terminals (6-OHDA+N, n = 6). The acquisition of a skilled forelimb reaching task was significantly impaired in 6-OHDA+D but not in 6-OHDA+N animals compared to vehicle (**Figure 1a** **,** *group×time* interaction: F(12,114) = 3.66; p<0.001, power 1.00; Bonferroni-corrected post hoc test between 6-OHDA+D and vehicle, p<0.001, power 1.00, and between 6-OHDA+D and 6-OHDA+N animals, p = 0.045, power 0.94). The slight reduction in reaching performance in 6-OHDA+N rats as compared with vehicle was not significant but the sample size was too small to conclusively evaluate the effect (p = 0.315). The time interval between reaching trials (pellet removal to subsequent door opening) was not different between groups (*time×group* interaction: p = 0.09, power 0.82) suggesting that the motivation was not affected by elimination of M1 dopaminergic terminals. Destroying M1 dopaminergic terminals at a time when the reaching task was already acquired had no effect on task performance (**Figure 1b**, n = 10, difference between day 11 and days 12-15, p = 0.87) indicating that dopamine is necessary for skill acquisition but not for movement execution.

**Figure 2:**
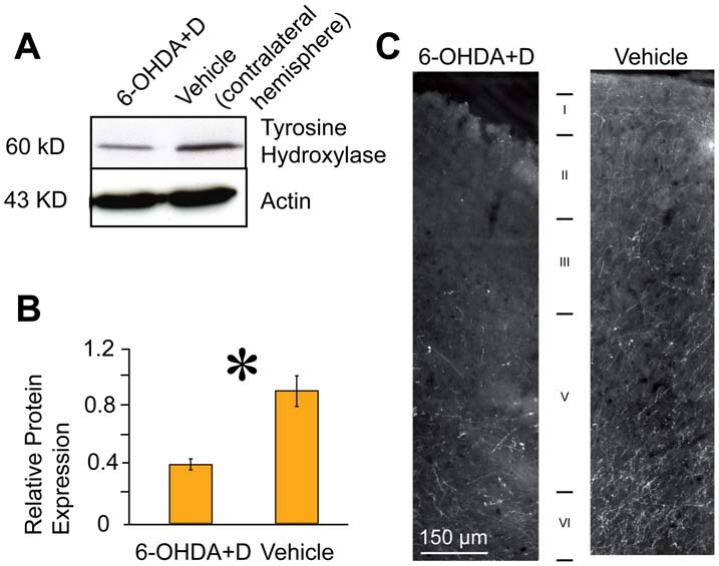
Identification of dopaminergic terminals in M1. (a) Western blot analysis of M1 cortical tissue injected with vehicle (sham-lesioned) and 6-OHDA in conjunction with desipramine (i.p.) using tyroxine hydroxylase (TH) reactivity indicated reduced TH expression after elimination of dopaminergic terminals. (b) Quantification of protein expression in DA-lesioned (6-OHDA+D) and sham-lesioned hemispheres reveals reduced protein expression after elimination of dopaminergic terminals. (c) Immunofluorescence staining of cortical dopaminergic terminals (TH immunoreactivity) in an exemplary vehicle and DA-lesioned hemisphere (6-OHDA injections into M1) indicated almost no staining in layer I and II/III and reduced staining in deeper layers in the lesioned M1. Similar findings were obtained in the other two animals treated analogously.

DA substitution using its precursor levodopa injected into the M1 forelimb representation reversed the 6-OHDA+D induced learning impairment. 6-OHDA+D and sham-lesioned rats were first trained for 6 days reproducing the findings described above in a new set of animals (**Figure 1c** **, phase 1**, interaction *group*×*time*: F(10,100) = 3.14, p = 0.002, power 0.98, post-hoc difference 6-OHDA+D+DA, n = 7, vs. vehicle+DA, n = 10: p<0.001, power 0.96, 6-OHDA+D+vehicle, n = 7, vs. vehicle+DA: p = 0.007, power 0.85, 6-OHDA+D+DA vs. 6-OHDA+D+vehicle: p = 1.00). Rats were then trained for six additional days after implantation of osmotic minipumps delivering either levodopa or vehicle into the M1 forelimb representation (**Figure 1c** **, phase 2**). Levodopa-treated rats reached control performance within 3 to 4 days while vehicle-treated rats did not show any performance improvement (interaction *group*×*time*: F(10,105) = 2.24, p = 0.021, power 0.90, post-hoc difference 6-OHDA+D+vehicle vs. vehicle+DA: p = 0.024, power 0.79, 6-OHDA+D+DA vs. vehicle+DA: p = 1.00, 6-OHDA+D+DA vs. 6-OHDA+D+vehicle: p = 0.13). Successful recall of the skill 6 days later did not require levodopa (**Figure 2c** **, phase 3**, differences, t-tests Bonferroni-corrected for 3 comparisons, 6-OHDA+D+DA vs. 6-OHDA+D+vehicle: p = 0.049, power 0.72; vehicle+DA vs. 6-OHDA+D+DA: p = 0.46; vehicle+DA vs. 6-OHDA+D+vehicle: p = 0.11). 6-OHDA+D followed by levodopa treatment did not affect the animals' motivation and attention because the intervals between reaching trials were not different between groups (p = 0.55). They rather slowly decreased during the three phases of the experiment in all animals (effect of *time*: F(5,68) = 10.62, p<0.001, power 1.00) [27].

The surgical procedure to perform intracortical injections caused a transient impairment of motor function as assessed using an accelerated rotarod test. The deficit between sham-lesioned (vehicle, n = 4) and 6-OHDA-treated rats (n = 4) was not different (**Figure 1d**, *group×time* interaction: p = 0.61) suggesting that the impairment of motor function resulted from the surgical procedure and not from the drug itself. Rotarod performance was lower at 3 hr, 6 hr and 9 hr post-injection (post hoc tests: p<0.05, power >0.75) with a statistical trend of reduced performance at 24 hr (p = 0.069) as compared with baseline. Performance recovered to pre-injection levels within 48 hr (**Figure 1d**, post-hoc difference to baseline: p = 1.00; overall effect of *time* F(6,30) = 3.44, p = 0.011, power 0.88). To guarantee full recovery a 3-day post-injection period was allowed following surgery before training was continued.

Successful destruction of dopaminergic terminals in M1 by 6-OHDA+D injections was verified by immunostaining and western blot analysis for tyroxin hydroxylase (TH). TH expression was significantly reduced in the 6-OHDA-treated hemisphere (n = 4, 6-OHDA+D, 0.501±0.04) compared with the contralateral hemisphere (0.76±0.12, p = 0.049, power 0.75, **Figure 2a, 2b**). Immunofluorescence was nearly absent in layer I and II/III and reduced in deeper layers following DA terminal destruction (**Figure 2c**). In contrast, TH immunoreactivity after injection of 6-OHDA+N to eliminate noradrenergic terminals was not significantly reduced (n = 4, 0.54±0.08) as compared to the non-injected hemisphere (0.62±0.20, p = 0.35).

### Dopamine receptors in M1 and motor skill learning

**Figure 3:**
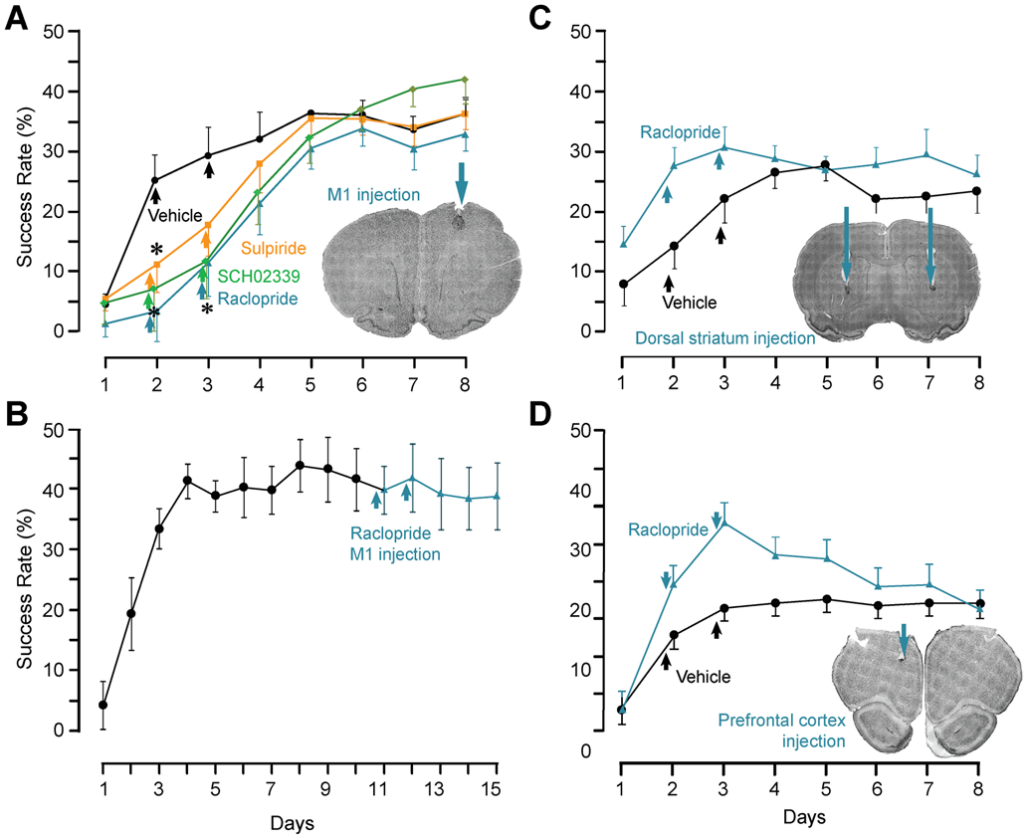
Functional D1 and D2 receptors in M1 are necessary for optimal motor skill acquisition but not for movement execution. (a) Blocking D1 receptors with SCH02339 (green) and D2 receptors with raclopride (blue) or sulpiride (orange) on the second and third day (arrows) of motor skill training significantly impaired reaching success compared to vehicle injected animals (black). When antagonists were discontinued, success rate began to increase normally. No significant differences in success rate existed at day 8 between all 4 groups. Inset: exemplary Nissl stain to verify cannula placement. (b) Raclopride injected into M1 (arrows) after the task had been acquired did not affect the performance. Inset: exemplary Nissl stain to verify injection cannula placement. (c,d) To exclude the possibility that the antagonists spread to other brain regions receiving important DA projections thereby causing the observed learning impairment, raclopride was injected into the dorsal striatum (c, blue) and the prefrontal cortex (d, blue) and compared to vehicle injected controls (black). Skill acquisition was not impaired in these animals. Insets: exemplary Nissl stain to verify cannula placement.

The type of DA receptor involved in motor skill learning was determined by application of specific antagonists. D2 receptors were temporarily blocked by injection of raclopride or sulpiride, and D1 receptors by injection of SCH23390 into the M1 forelimb area contralateral to the forelimb used for reaching. The success rate was significantly reduced in the presence of raclopride (n = 7), sulpiride (n = 6), and SCH23390 (n = 6) compared to vehicle-injected animals (n = 8) (**Figure 3a**, *time×group* interaction: F(11,99) = 2.54, p = 0.001, power 1.00, post-hoc Dunnett's tests for comparisons with the control group: raclopride p = 0.033, power 0.86, sulpiride p = 0.012, power 0.73, SCH23390 p = 0.040, power 0.72). Reaching performance increased upon discontinuation of the antagonists and reached the level of controls within three days. Similar to animals in which dopaminergic terminals were eliminated in M1, motivation and attention were not affected by D1 or D2 antagonists (*group×time* effect on intertrial intervals: p = 0.98). Raclopride did not alter the execution of the previously acquired skill (**Figure 3b**, n = 5, effect of *time*: F(14,42) = 16.97, p<0.001, power 1.00; post-hoc testing: no significant difference between session 10 and the average of sessions 11-15, p = 0.41). These results suggest the involvement of D1 and D2 receptors in motor skill acquisition.

Antagonist effects on skill learning were not indirectly caused by a spread of the drug to neighboring brain regions known to receive dopaminergic input. Raclopride injected into the dorsal striatum (n = 10, **Figure 3c**) and the prefrontal cortex (PFC, n = 6, **3d**) during motor skill training did not significantly affect learning curves as compared to vehicle (n = 10 for striatum, n = 6 for PFC; interaction *group×time*: striatum F(7,98) = 0.57, p = 0.78; PFC F(7,63) = 1.12, p = 0.36).

### Synaptic transmission and plasticity

**Figure 4:**
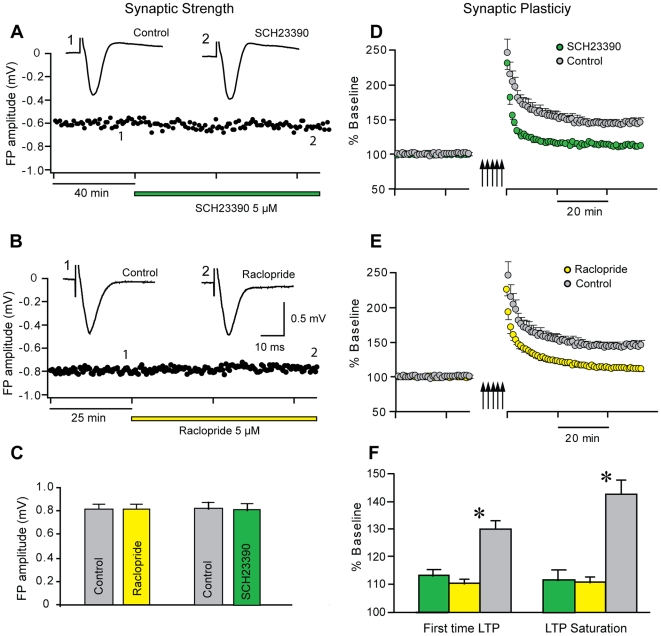
Synaptic plasticity but not synaptic transmission depends on DA receptor activity in M1. (a,b) Exemplary time courses of peak amplitudes of extracellular field potentials (FP) in layer II/III horizontal connections in the M1 forelimb area recorded in brain slices. FP amplitudes at baseline stimulation intensity before (control) and after D1 (a, SCH02339, green) and D1 receptor blockade (b, raclopride, yellow). Antagonists do not modify amplitude or shape of FPs. Insets: each trace represents an average of 10 individual traces at times indicated by numbers. (c) Group data indicate no significant difference before (control) and after antagonist application. (d, e) LTP was induced repeatedly (multiple arrows) until responses were saturated in normal ACSF (control, grey) and in the presence of SCH02339 (d, green) or raclopride (e, yellow). (f) Group data show significantly reduced LTP in the presence of D1 and D2 receptor antagonists compared to controls (grey) for single LTP induction (left) and saturated LTP (right). In the presence of DA antagonists responses are already saturated after the first LTP attempt.

Previous studies have shown that motor skill learning enhances synaptic strength and partially occludes long term potentiation (LTP) in layer II/III and layer I of the M1 forelimb area [24], [25] suggesting that LTP strengthens synapses during motor skill learning. Since blocking dopaminergic transmission in M1 during skill acquisition impairs learning we hypothesized that the learning impairment might be accompanied by an impairment of LTP. Therefore, we examined synaptic transmission and synaptic plasticity in cortical slices before and after DA antagonist application. Amplitudes of extracellular field potentials (FP) in layer II/III of coronal slices were not affected by D2-receptor antagonist raclopride (**Figure 4a, c** **,** 0.814±0.050 mV vs. vehicle: 0.818±0.048 mV, n = 10, p = 0.954) or D1-receptor antagonist SCH23390 (**Figure 4b, c** 0.822±0.054 mV vs. vehicle: 0.827±0.056 mV, n = 22, p = 0.952). However, the capacity for synaptic plasticity was reduced when D1 or D2 receptors were blocked. After establishing baseline responses, LTP was induced repeatedly until saturated. LTP was significantly reduced in the presence of raclopride or SCH23390 (raclopride: 110.99±1.67%, n = 12, SCH23390: 111.91±3.99%, n = 10) compared to control conditions (142.74±5.2%, n = 8; effect of *group*: F(2,27) = 23.6, p<0.001, power 1.00; Bonferroni-corrected post-hoc difference: raclopride vs- vehicle: p<0.001, power 1.00, SCH 23390 vs. vehicle: p<0.001, power 1.00, raclopride vs. SCH 23390: p = 1.00, power 0.05, **Figure 4d-f**). With DA receptors blocked responses were already saturated with a single LTP attempt (raclopride: 110.65±1.42%, n = 15; SCH23390: 113.51±2.35%, n = 11) while 2 to 4 attempts were required in control condition (vehicle: 130.25±2.93%, n = 13, **Figure 4f**). These results show that dopaminergic transmission is necessary for optimal expression of LTP.

## Discussion

We found that dopaminergic signaling in primary motor cortex (M1) is necessary for normal motor skill learning and synaptic plasticity within M1, but not for the execution of a learned task or for synaptic transmission.

Neither destruction of M1 dopaminergic terminals nor dopamine receptor antagonists abolished learning completely. DA in M1, therefore, seems to act as a modulator of motor skill learning that enables optimal task acquisition. Other modulators, such as acetylcholine [28], [29], serotonin [30] and GABA [31], may be able to partially compensate for the lack of DA by increasing (or decreasing in the case of GABA) their activity restoring some but not optimal learning.

Recall and execution of the learned movements were intact in the absence of dopaminergic signaling suggesting that recall and use of stored motor information does not require DA in M1. This result does not contradict the well-known influence of central nervous system DA on movement execution. DA affects behavior depending on the brain region where it is released. For example, injections of 6-OHDA into the basal ganglia produce marked alterations in reaching movements in rats [32]. Our results reveal no such effects after interfering with dopaminergic transmission in M1. Instead, we show that DA in M1 is specifically important for the formation of new motor memories.

M1 is one of the brain regions critically important for motor skill learning. Eliminating protein synthesis in M1 abolishes the learning of reaching skills in rats [33]. Asanuma and colleagues showed that LTP can be induced in M1 by tetanic stimulation of the somatosensory cortex or by associative stimulation of somatosensory cortex and thalamus [34]. Considering that motor learning is impaired after lesioning relevant regions of somatosensory cortex [35], [36], it is assumed that skill learning is in part mediated by synaptic plasticity in M1 that is driven by somatosensory input [37]. Motor skill learning also enhances synaptic responses of intracortical connections in M1 [23], occludes LTP in these connections [24] and induces structural modification of M1 dendritic spines [25]. Here, we show in brain slices that blocking DA receptors reduces the capacity of horizontal M1 connections to express LTP. This finding cannot be explained by a remote dopaminergic effect but is rather the result of directly blocking DA receptors in M1, because in the slice, remote inputs from other brain regions, such as the basal ganglia, are transected. LTP in M1 horizontal connections has been linked to learning of a skilled reaching task [24] and may therefore be one mechanism by which DA modulates motor skill learning. In prefrontal cortex (PFC) DA antagonists similarly reduce LTP [16] and impair learning and memory [38], [39]. Our results emphasize the role of DA as an essential neurotransmitter involved in cortical plasticity and learning.

D1 and D2 receptors are generally known to have opposing effects in individual neurons by increasing and decreasing cAMP production, respectively [39]. We found that D1 and D2 receptor antagonists in M1 had similar effects on learning and synaptic plasticity. They both impair motor skill learning and LTP formation. These findings seem contradictory to the opposing intracellular effects of D1 and D2 receptors. Concordant actions of both antagonists in M1 have been described before. Spontaneous firing of M1 pyramidal neurons is increased by both D1 and D2 receptor antagonists [40]. Concordant actions could be explained by a network effect assuming that D1 and D2 receptors – with opposing intracellular actions – are located on excitatory and inhibitory neurons leading to a common net effect on M1 output. In support of this hypothesis, D1 and D2 receptors have been found to be distributed differently across the layers of M1 [41]. Alternatively, the receptors may exist in alternative configurations not linked to cAMP production. A dimeric configuration of both receptors activating phospholipase C has been reported [42]. Phospholipase C increases intracellular Ca^2+^ [43] a potent stimulant for learning and memory [44].

Several methodological issues warrant a critical discussion. Spread or diffusion of drugs injected into M1 could have resulted in dopamine depletion or blockade in other brain regions thereby confounding the behavioral observations. In a previous study we have evaluated our injection technique and have found no evidence for spread or diffusion outside of M1 [33]. Here, we injected raclopride into striatum and PFC, two regions receiving dense dopaminergic projections, and found no evidence for a motor learning impairment. This does not contradict the involvement of striatum and PFC in motor learning due to their roles in motor control and attention, respectively. It shows that using identical injections at identical time points for all three regions, learning is only impaired after motor cortex injections. Because the effect of a direct injection should be more prominent than indirect spread or diffusion, false positive results of motor cortex injections are highly unlikely.

Also intertrial latencies remained unaffected in all our experiments strengthening the argument for the specificity of M1-dopaminergic influence on motor skill learning. Latencies would increase if animals were distracted, not motivated or failed to remember the spatial and conceptual requirements of the task (how to open the door, where to find the pellet).

Given previous reports of learning deficits after DA depletion in striatum [32], it seems unexpected that raclopride injected into striatum had no effect on learning. However, we have used much lower concentrations and smaller injection volumes most likely leading to less spread of drug, hence, affecting less striatal tissue and different parts of the striatum. Interfering with motor behavior requires striatal dopamine depletion to pass a certain threshold as demonstrated by assessing forelimb use symmetry after different doses of 6-OHDA injected into the striatum [45]. Such a threshold most likely exists also for the tissue volume affected: Miklyaeva et al. [32] observed learning impairments after injecting 8 µg 6-OHDA dissolved in 4 µl. Injections restricted to the medial striatum had less effect on reaching than those to the lateral striatum [46]. We injected only 0.5 µl of a raclopride solution affecting smaller volumes of tissue. Injection volume was chosen to be identical to cortical injections with the purpose to exclude the possibility that learning deficits are indirectly caused by raclopride spreading or diffusing into adjacent brain regions where dopamine acts as a neurotransmitter.

Our findings offer a mechanism to explain observations that were previously not understood. In humans learning a motor skill, measurements of DA metabolites in the cerebrospinal fluid correlate with the learning rate [7]. Motor skill learning and plastic adaptation in the M1 is improved by systemic administration of levodopa [6]. Our finding that M1 dopaminergic signaling is important for motor skill learning and synaptic plasticity could explain accelerated movement recovery after brain injury when motor training is combined with systemic levodopa administration [1]; plasticity in M1 is a candidate mechanism to support movement recovery after brain injury [2], [47] and this plasticity my be facilitated by levodopa. In patients with Parkinson's disease dopaminergic neurons degenerate in the substantia nigra (SN) and the ventral tegmental area (VTA) [48]. It is well known that loss of dopaminergic neurons projecting from the SN to the striatum causes the cardinal symptoms of akinesia, tremor and rigidity. Dopaminergic projections from the SN to cortex have not been reported [49], [50]. However, the VTA contains dopaminergic neurons projecting to frontal cortex and some evidence exists that some VTA neurons send axons to M1 [reviewed in 51]. Therefore, patients with Parkinson's disease may very well have deficient dopaminergic projections from VTA to M1. Considering that these patients show reduced M1 plasticity [52] and deficits in skill [8] and procedural learning [53], our findings provide a potential mechanism for these deficits, that is the degeneration of M1 dopaminergic signaling.

In conclusion, dopaminergic neurotransmission in primary motor cortex plays a crucial role for motor skill learning and synaptic plasticity in M1. These findings may open opportunities for the development for novel therapies aiming to restore motor function after brain injury or motor deficits in Parkinson's disease.

## Materials and Methods

All experiments were performed in adult male Long-Evans rats (8–10 weeks, 250–350 g) raised in our animal facility. Animals were housed individually in a 12/12-hr light/dark cycle (light on: 3am, off: 3pm). All procedures were conducted according to national and international guidelines and were approved by the Animal Care Committee of the State of Baden-Württemberg, Germany. Chemicals and antibodies were purchased from Sigma-Aldrich Chemie GmbH, Munich, Germany, unless noted otherwise.

### Behavior

#### Motor skill training

Training sessions were performed at the beginning of the dark phase. Animals were food-restricted for 24 hr before the first pre-training session (see below). During training animals were kept slightly over their initial weight (332.1±29.4 g) by providing 50 mg/kg of standard lab diet after each training session. Water was given *ad libitum*.

Motor skill training was performed as previously described [27]. The training cage was a 15×40 cm chamber (height 30 cm) with a vertical window (1 cm wide, 5 cm high beginning 2 cm above floor) in the front wall and a small light sensor in the rear wall (7 cm above ground). Animals were first pre-trained for five days learning to open the motorized sliding door that covered the front window, by nose-poking the sensor in the rear. Opening the window gave access to one food pellet (45 mg, Bio-serve, Frenchtown, NJ, USA) located on a small horizontal board outside of the cage. During pre-training pellets were retrieved by tongue. Upon retrieval the pellet was automatically replaced by a pellet dispenser. Pre-training was followed by 6–15-days of motor skill training that was initiated by removing the board and placing the pellet on a vertical post 1.5 cm away from the window. In this position pellets were only retrievable by using the forelimb. The first skill training session was to determine forelimb preference and consisted of 50 door openings ( = trials). Determination of preference was necessary before surgical instrumentation of the hemisphere contralateral to the preferred limb (see below). After determining forelimb preference, the pedestal was shifted to one side of the window to allow reaching with the preferred limb only, contralateral to the hemisphere injected or instrumented for drug injection. All subsequent sessions consisted of 100 trials. The movement required to retrieve the pellet consisted of a forelimb extension to the target, followed by pronation, paw opening, grasping motion, forelimb retention combined with a supination to bring the pellet to the mouth. Rats mainly improved target reach and grasp elements to successfully retrieve the pellet during training sessions. Each reaching trial was scored as “successful” (reach, grasp and retrieve) or “unsuccessful” (pellet pushed off pedestal or dropped during retraction). The success rate was defined as the ratio of the number of successful trials and the total number of trials per session, i.e. 100. The latency between pellet removal and subsequent door opening was used as an index of motivation [27]. Daily training sessions consisted of 100 trials ( =  door openings), involved 115.8±5.7 forelimb movements, mean±SEM, automatically sensed by a sensor between cage wall and pedestal) and lasted 24.8±0.5 min, mean±SEM.

#### Rotarod test

Four-limb motor function was examined to avoid confounds of post-surgical discomfort and potential motor deficits, and was assessed using an accelerated rotarod test (7 cm diameter rod accelerating at 1 cm/s^2^). Maximum velocity at the time the rat fell off the rod, was an index of four-limb motor function. Twenty runs were performed per session with a 15 sec break between runs. Because rotarod performance initially improves with practice [54], two baseline sessions were completed.

### Surgical Procedures

All surgical procedures were performed under ketamine (70 mg/kg, i.p.) and xylazine anesthesia (5 mg/kg, i.p.) with the rats fixed in a stereotactic frame (Stoelting Co., Wood Dale, IL, USA). Additional ketamine doses were administered if necessary. Body temperature was controlled using a heating pad. Buprenorphin (0.01 mg/kg, i.p.) was given after surgery for pain relief. All permanent implants were anchored onto the skull with two screws (2 mm diameter) placed in the frontal and occipital skull. Bone flaps were replaced and fixated using bone cement (FlowLine, Heraus Kulzer, Dormagen, Germany).

#### Identification of the M1 forelimb area

The forelimb representation was identified in each animal for optimal placement of injection needles (6-OHDA and levodopa) and cannula implantation (repeated antagonist injections). The brain was exposed by craniotomy leaving the dura intact (coordinates with respect to Bregma: 4 mm posterior to 5 mm anterior, 5 mm to 1 mm lateral). M1 somatotopy was mapped using a thin-film microelectrode array (Multichannel Systems, Reutlingen, Germany) as previously described [55]. The array was placed onto the dura over the frontoparietal cortex. Biphasic stimuli (100 stimuli at 300 Hz, 1–5 mA constant current, 10 ms stimulus interval) were applied to the 64 contacts of the electrode array in a random sequence. Evoked limb twitches were visually identified. The forelimb area was typically 2 to 3.5 mm lateral and 1.5 to 2.5 mm anterior to Bregma. All drug injections were performed in a depth of 1 mm below the dura.

### Histology

Positioning of guide cannulas for antagonist injections and positioning of needles for single injections of 6-hydroxydopamine (6-OHDA), and continuous infusion of levodopa with osmotic minipumps was verified in each animal histologically by Nissl staining. No animal had to be excluded because of misplacement of needles or cannulas.

### Elimination of dopamine terminals in M1

To test whether elimination of dopaminergic terminals in M1 affects motor skill learning, animals were injected with 6-OHDA into M1 (0.5 µl of 6 µg/µl 6-OHDA in 0.1% ascorbic acid, single injection without guide cannula to minimize cortical injury). At the same time, desipramine (20 mg/kg, i.p.) was administered to protect noradrenergic neurons (6-OHDA+D, n = 6). These animals were compared with sham-lesioned controls (0.5 µl of 0.1% ascorbic acid in 0.9% NaCl, vehicle, n = 11) and animals with selective elimination of noradrenergic terminals using intracortical 6-OHDA injections plus nomifensine (10 mg/kg, i.p., 6-OHDA+N, n = 6). All injections were performed after training day 1 (50 trials) to determine forelimb preference and baseline performance.

To test whether 6-OHDA+D effects were specific for motor skill acquisition or were the result of a movement execution deficit, the drug was injected during the plateau phase of the learning curve, when no further skill learning occurred (injection on day 11, n = 10).

### Levodopa substitution

In separate groups of rats, levodopa (5 mg/ml plus 1.25 mg/ml benserazide, dissolved in 0.9% NaCl, containing 0.2 mg/ml ascorbic acid) was administered into the M1 of rats with destroyed M1 dopaminergic terminals to test whether levodopa can restore the ability to acquire the motor skill. After the baseline training session (50 trials), 6-OHDA+D lesions were performed. Following a 3-day recovery period, rats were trained for 6 days. Then, osmotic minipumps (0.25 µl/hr, 100 µl volume, model 1002, Alzet, Cupertino, CA, USA) filled with levodopa or 0.9% NaCl were implanted subcutaneously into the neck area. Minipumps were connected by tubing tunnelled under the skin to a needle implanted into the M1 forelimb representation. Animals recovered for 3 days and were then retrained for 6 days. After the last training session minipumps were explanted and recall training was performed following 6 days of rest. 6-OHDA+D-lesioned and levodopa-infused animals (6-OHDA+D+DA, n = 7) were compared with 6-OHDA+D-lesioned rats infused with vehicle (0.9% NaCl containing 0.2 mg/ml ascorbic acid, 6-OHDA+D+vehicle, n = 7) and with sham-lesioned rats implanted with levodopa-containing minipumps (vehicle+DA, n = 10).

### Immunochemical assessment dopamine terminals in M1

#### Western Blot analysis

The presence of dopaminergic terminals in M1 was assessed using tyrosin hydroxylase Western Blot analysis. One hemisphere was injected with 6-OHDA, the contralateral hemisphere with vehicle (0.5 µl of 0.1% ascorbic acid in 0.9% NaCl). In addition, rats received desipramine (n = 4, 20 mg/kg, i.p.) to protect noradrenergic neurons while others received nomifensine to protect dopaminergic neurons (n = 4, 10 mg/kg, i.p.). After three days rats were decapitated in ether anesthesia, brains were quickly dissected over ice to isolate cortex, and visually inspected to verify the injection site. The cortex was frozen on dry ice. Tissue samples were sonicated in lysis buffer containing a protease inhibitor cocktail, centrifuged, and the supernatant extracted for gel electrophoresis. Equal amounts of lysates were subjected to SDS-PAGE gels, which were then transferred to nitrocellulose membranes. After blocking with 5% skim milk in tris-buffered saline, blots were incubated with primary antibody, tyrosine hydroxylase (1∶1000, Chemicon, Hofheim, Germany). Antibody binding was detected using a horseradish peroxidase secondary antibody (Amersham Biosciences, GE Healthcare, Munich, Germany) and enhanced chemiluminescence. Membranes were then stripped for 15 min at 25°C with Stripping Buffer (Pierce, Rockford, IL, USA) and sequentially re-probed with polyclonal anti-β-actin antibody (1∶1000, Santa Cruz, Heidelberg, Germany) to ensure equal protein loading across samples. Exposed films were scanned and analyzed using band densitometry (Scion Image software, Frederick, MD, USA).

For **immunohistochemistry**, three rats were treated with 6-OHDA, vehicle and desipramine as described above. After three days, the animals were perfused transcardially with 4% paraformaldehyde in 0.1 M phosphate buffer (PB, pH 7.4) after inducing deep anesthesia (pentobarbital, 50 mg/kg i.p.). Brains were kept for 24 hr in 4% PFA and then for 3–4 days in 30% sucrose before rapid freezing in 2-methyl buthan. Coronal sections (40 µm) were prepared using a cryostat (Leica Microsystems GmbH, Wetzlar, Germany). Sections were rinsed twice in PBS, treated with 0.1% Triton in PBS for 10 min and blocked for 30 min in 10% goat serum. The sections were first incubated in a primary antibody dilution (tyrosine hydroxylase, 1∶100) in PBS, for 24 hr at 4°C, then in secondary antibody dilution (cy3-conjugated) at room temperature for 1 hr, and finally in Hoechst stain for 10 min. Sections were mounted with Mowiol and inspected under a fluorescent microscope (Axioplan II, Zeiss AG, Jena, Germany).

### Dopamine antagonists in M1

To test the effects of DA receptor antagonist administration in M1 on motor skill learning, rats received intracortical injections of the D2-receptor antagonists raclopride (n = 7, 10 µg/µl, S(−)-Raclopride (+)-tartrate salt in 0.9% NaCl) or sulpiride (n = 6, 20 µg/µl, (S)-(−)-sulpiride in 0.9% NaCl), or the D1-receptor antagonist SCH 23390 (n = 6, 600 µg/µl in 0.9% NaCl, Tocris Biosciences, Avonmouth, UK), or vehicle solution (n = 8, 0.9% NaCl) 30 min before the beginning of the training sessions on day 2 and 3 of an 8-day training period. Rats were implanted with a guide cannula (15 mm long, Unimed SA, Lausanne, Switzerland) into the center of the left M1 forelimb representation. Cannulas allowed drug application while the investigator restrained the rat during injection *in lieu* of anesthesia. For injection a needle (34 Gauge, same length as guide cannula, Hamilton Bonaduz AG, Switzerland) was inserted into the guide cannula after removing the obturator. A volume of 0.5 µl was injected over 90 sec using a microsyringe (5 µl, Hamilton Bonaduz AG, Switzerland) connected via tubing (10 cm, PE40 Plastics One, Roanoke, VA USA) to a microinjection pump (Nano-injector, Stoelting Co., Wood Dale, IL, USA).

To test whether raclopride effects were specific for motor skill acquisition or were the result of a movement execution deficit, the drug was injected during the plateau phase of the learning curve, when no further skill learning occurred (injections before training on days 11 and 12, n = 5).

To test whether drug injections were specific to M1 and did not spill into adjacent areas known to receive dopaminergic projections, raclopride (10 µg/µl, 0.5 µl) or vehicle (0.9% NaCl) were injected into the dorsal striatum (implantation coordinates 3 mm lateral, 0.5 mm anterior to bregma, 5 mm below dura, raclopride n = 10, vehicle n = 10) or prefrontal cortex (coordinates: 1 mm lateral, 5 mm anterior to bregma, 1 mm below dura, raclopride n = 6, vehicle n = 6).

### Dopamine antagonist effects on synaptic transmission and plasticity

#### In vitro slice preparation

Deeply anesthetized untrained rats (pentobarbital, 50 mg/kg) were decapitated, their brains quickly removed and immersed in cold (5–7°C), oxygenated (95% O_2_/5% CO_2_) artificial cerebrospinal fluid (ACSF) containing (in mM): 126 NaCl, 3 KCl, 1.25 NaH_2_PO_4_, 1 MgSO_4_, 2 CaCl_2_, 26 NaHCO_3_, 10 dextrose. Coronal slices (500 µm) including the M1 forelimb area (1.5–3.5 mm anterior to Bregma, 2–4 mm lateral) of both hemispheres were prepared using a vibratome. Slices were transferred to a temperature controlled (34±0.5°C) interface chamber and superfused with oxygenated ACSF at a rate of 1–2 ml/min. Slices were allowed to recover for at least one hour.

#### Stimulation and field potential recording

Concentric bipolar stimulation electrodes were positioned symmetrically in layer II/III of each hemisphere 2–2.5 mm lateral to the midline, and recording electrodes placed 500 µm laterally. Extracellular field potentials (FP) were evoked by 0.2 ms, 0.03 Hz stimulation and recorded simultaneously in both hemispheres. Stimulation intensity was adjusted until a response of 0.2 mV was recorded, which was defined as the threshold intensity. Multiples of this intensity were used for determination of input-output relationships to assess the baseline synaptic strength.

#### Induction of LTP

The stimulus intensity eliciting 50% of the maximum amplitude was used for all measurements before and after LTP induction. Baseline amplitudes were recorded using single stimuli applied every 30 sec. Following a 30-min stable baseline period, LTP was induced by theta burst stimulation (TBS), consisting of 10 trains of 5 Hz stimuli, each composed of 4 (200 µsec) pulses at 100 Hz, repeated 5 times every 10 sec. During TBS the stimulation intensity was doubled. TBS was applied immediately after transient, local application of the GABA_A_ receptor antagonist bicuculline methiodide (3.5 mM) at the field potential recording site until response amplitude increased to 150–200% of baseline.

### Statistical Analysis

Data analysis was performed using Statistica version 7.0 (StatSoft Inc., Tulsa OK, USA). Reaching performance was quantified as the percentage of trials with successful retrievals per session ( = 100 trials). General linear repeated measures models were used to test for effects of *training day* on *reaching performance* including *group, baseline performance*, and the interaction of *group×time* (session) as independent variables. Whether data met the sphericity condition was tested using Mauchly's criterion and if not met, Geisser and Greenhouse correction was applied. Post hoc tests were performed using Bonferroni correction for multiple comparisons or Dunett's test for comparison with a control group. Two-tailed probability less or equal to 5% was considered significant.
